# Pathogenesis and Management Strategies in Radioiodine-Refractory Differentiated Thyroid Cancer: From Molecular Mechanisms Toward Therapeutic Approaches: A Comprehensive Review

**DOI:** 10.3390/jcm13237161

**Published:** 2024-11-26

**Authors:** Iulia-Alexandra Voinea, Eugenia Petrova, Nicoleta Dumitru, Andra Cocoloș, Dumitru Ioachim, Andrei Liviu Goldstein, Adina Mariana Ghemigian

**Affiliations:** 1 PhD Doctoral School, “Carol Davila” University of Medicine and Pharmacy, 0505474 Bucharest, Romania; 2 Department of Endocrinology, “Carol Davila” University of Medicine and Pharmacy, 020021 Bucharest, Romania; eugenia.petrova@umfcd.ro (E.P.); nicoleta.dumitru@umfcd.ro (N.D.); andra_buruiana@yahoo.com (A.C.); adina.ghemigian@umfcd.ro (A.M.G.); 3 Department of Clinical Endocrinology V, C.I. Parhon National Institute of Endocrinology, 011863 Bucharest, Romania; 4 Department of Pathology, C.I. Parhon National Institute of Endocrinology, 011863 Bucharest, Romania; 5 Department of Nuclear Medicine, C.I. Parhon National Institute of Endocrinology, 011863 Bucharest, Romania; andrei_goldstein@yahoo.com

**Keywords:** differentiated thyroid cancer, RAIR-DTC, NIS, signal pathways, tyrosine kinase inhibitors, surgery, iodine therapy

## Abstract

Thyroid cancer (TC) remains the most common cancer in endocrinology. Differentiated thyroid cancer (DTC), the most common type of TC, generally has a favorable outlook with conventional treatment, which typically includes surgery along with radioiodine (RAI) therapy and thyroid-stimulating hormone (TSH) suppression through thyroid hormone therapy. However, a small subset of patients (less than 5%) develop resistance to RAI. This resistance occurs due to the loss of Na/I symporter (NIS) activity, which is crucial for iodine absorption in thyroid cells. The decline in NIS activity appears to be due to gene modifications, reconfigurations with irregular stimulation of signaling pathways such as MAPK and PI3K/Akt pathways. These molecular changes lead to a diminished ability of DTC cells to concentrate iodine, which makes RAI therapy ineffective. As a consequence, patients with radioiodine-refractory DTC require alternative treatments. Therapy with tyrosine kinase inhibitors (TKIs) has emerged as the primary treatment option to inhibit proliferation and growth of RAIR-DTC, targeting the pathways responsible for tumor progression. In this article, we analyze molecular processes responsible for RAI resistance and explore both conventional and emerging therapeutic strategies for managing RAIR-DTC, aiming to improve patient outcomes.

## 1. Introduction

Thyroid cancer (TC) is the most common endocrine tumor, and in most cases it is a differentiated thyroid cancer (DTC) [1,2]. Less than 5% of the subjects with poorly DTC have resistance to radioactive iodine therapy; thus, poor prediction is found, with an average life expectancy of 3–5 years. A multidisciplinary approach is needed to establish a personalized strategy [3,4].

The most prevalent forms of TC are papillary and follicular, making up over 90% of cases and collectively classified as DTCs [5]. These DTCs are usually slow-growing and have a favorable prognosis, with survival lasting 20 years or more after standard treatment [6,7].

For most patients with DTC, standard treatment—typically involving surgery followed by radioiodine (RAI) ablation, risk-adjusted monitoring, and thyroid-stimulating hormone (TSH) suppression treatment—is generally effective [8]. Nevertheless, local recurrence and distant metastases can occur in approximately 20% and 10% of patients, respectively, within the first decade after surgery [6]. Traditional treatment options for such cases include repeated radioiodine treatment, surgical treatment of metastases, and external radiation. Despite these interventions, about two-thirds of DTC cases eventually become resistant to RAI therapy, which worsens prognosis and life expectancy [1]. Once resistance develops, the survival rate at 10 years drops to about 20% [9].

In recent years, progress in genome sequencing has greatly improved our comprehension of the molecular pathways behind TC [3]. Most of the TC in this subgroup show alterations in the MAPK and PI3K/mTOR/Akt signal pathways, which are essential for regulating cell growth and division by transmitting signals from the cell membrane to the nucleus [10,11].

The excessive activation of the MAPK pathway plays a key role in the onset of papillary TC (PTC), often resulting from base substitutions in the *BRAF* oncogene. *BRAF*, part of the RAF family of serine/threonine kinases that is downstream of *RAS*, is commonly altered in PTC, associated with mutation rates reported between 29% and 83% [12,13,14,15]. This mutation triggers transcription factors that drive processes like cell expansion, maturation, cell division, and programmed cell death. While various pathogenic *BRAF* variants were identified, the *BRAF*^V600E^ mutation is the most common in classic PTC cases [16]. Research has linked this variant to more aggressive disease characteristics, such as metastasis, invasion, and recurrence [17]. *BRAF^V600E^* mutation also promotes modulating TGF-β production, which suppresses sodium iodide symporter (NIS) expression, resulting in resistance to RAI therapy [18].

Similarly, stimulation of PI3K/mTOR/Akt signaling cascade is crucial during follicular TC (FTC) development. This pathway is activated due to mutations in the RAS, PIK3CA, and AKT1 oncogenes or due to the loss of function of the PTEN oncogene, which normally plays the part of a negative regulator. *RAS* pathogenic variants, which drive both MAPK and PI3K-Akt signaling cascades, are commonly observed in FTC cases (between 28 and 68%), as well as in as many as 43% of the follicular-variant PTCs and 47% of non-invasive follicular-variant PTCs [19,20]. However, *RAS* pathogenic variants alone appear to have a relatively limited effect on clinical outcomes in TC [21].

As TC advances and loses differentiation, transforming into poorly DTC or anaplastic thyroid cancer (ATC), additional pathogenic variants—such as those affecting the p53 and Wnt/β-catenin pathways—become involved. Recent research has also discovered alterations in *TERT* promoter across every single TC variant, more frequently found in aggressive and poorly DTC, underscoring their role in driving disease development [22,23,24].

As our understanding of the genetics of malignant thyroid disease advances, treatment approaches have evolved from concentrating solely on tumor type and histological features to targeting specific genetic alterations. This shift has resulted in the creation of new targeted therapies aimed at patients with more aggressive forms of the disease [25,26].

### Objective

This review outlines the molecular process behind TC that contributes to refractoriness to RAI in DTC, as well as the modern diagnostic and treatment management strategies. We highlight the specific genetic alterations associated with this resistance and examine both conventional and emerging therapeutic approaches, including targeted therapies and innovative strategies to address treatment challenges. Additionally, we discuss the importance of precision medicine in optimizing patient outcomes and enhancing the effectiveness of existing treatment options.

## 2. Materials and Methods

An exhaustive search was made using PubMed, Scopus, and Google Scholar to identify recent studies and guidelines focused on the molecular mechanisms and the management of diagnosis and treatment for radioiodine-refractory DTC (RAIR-DTC). The search is a narrative comprehensive review and targeted English-language publications from 1998 to 2024, with a focus on both experimental and clinical trials. The search strategy incorporated key terms such as “differentiated thyroid cancer”, “RAIR-DTC”, “NIS”, “signal pathways”, “tyrosine kinase inhibitors”, “surgery”, and “iodine therapy”.

Table 1 lists the inclusion and exclusion criteria that formed the basis of this review.

## 3. Pathogenesis of RAIR-DTC

Resistance to RAI occurs as a result of losing thyroid differentiation. Dedifferentiation is a consequence of damage to the NIS. Part of SLC5A5, NIS is a basolateral membrane glycoprotein in follicular epithelial cells. Iodine, as a necessary component in the follicular synthesis of thyroid hormones, enters the cell actively through NIS. Normally, NIS transcription begins when TSH binds with the TSH receptor and the cAMP pathway is immediately initiated. Then, cAMP enhances some activating pathways that contribute to NIS upstream enhances (NUE) stimulation. Thus, this stimulation of NUE is performed either in a PKA-dependent or PKA-non-dependent manner. For the case of the independent PKA pathway, Paired box gene-8 (PAX8) is activated using Ref-1, thus linking to NUE. This mechanism has a key role in the process of follicular cell differentiation. Regarding the PKA-dependent route, aAMP-response element modulator (CREM) amplifies the NUE function [27,28,29].

The decrease in the NIS signal, which is responsible for resistance to RAI, appears as a result of modulation of signaling pathways, chromosomal rearrangements, or aberrant gene methylation [27,29,30].

### 3.1. Molecular Genetic Characterisation

#### 3.1.1. BRAF Pathogenic Variant and Rearrangement

*BRAF*, which is a proto-oncogene belonging to a family of serine/threonine kinases, has fundamental importance of MAPKKK in the MAPK signaling cascade [30]. T1799A point genetic alteration located in exon 15 is one of the most common mutations in the *BRAF* gene [31]. This missense mutation leads to a change in B-raf protein residue 600, replacing glutamic acid with valine (V600E) and the persistent serine/threonine kinase function that damages the suppression loop. As a result, *BRAF^V600E^* could initiate itself and also the MAPK signaling cascade [30,32].

Undoubtedly, *BRAF^V600E^* remains the most common genetic mutation in thyroid cancers, being described in more than half of the DTCs [27]. According to clinical studies, patients with PTC with *BRAF^V600E^* would have good prognoses. The synergistic action of *BRAF^V600E^* with another gene mutation increases the aggressiveness. Studies suggest that patients with PTC and *BRAF^V600E^* pathogenic variant develop aggressive pathological features, high risk of recurrence, and lack of RAI capture. The co-association of *BRAF^V600E^* and *CYP2S1* adversely affects PTC. The presentation of *CYP2S1* is controlled by the MAPK signaling pathway mediated by *BRAF^V600E^* with the help of the AHR-dependent cascade. The AHR/CYP2S1 feedback mechanism increases the impact of mutations on BRAFV600E. Moreover, the *BRAF^V600E^* proto-oncogene can be connected with Wilm tumor gene1 (*WT1*), which has a function in transcription of a gene that is important for cell viability, differentiation, as well as proliferation [30,33,34].

*BRAF* fusion is an additional critical factor that determines TC progression. According to a study conducted on 65 Ukrainian-American individuals with PTC subjected to the effects of Chernobyl radiation, several alterations in MACF-BRAF, MBP-BRAF, and POR-BRAF were discovered through next-generation sequencing (NGS) and RNA sequencing. These may be responsible for the evolution of TC with radiation exposure [35].

#### 3.1.2. NTRK Gene Fusion

*NTRK* (such as *NTRK1*, *NTRK2,* and *NTRK3)*, is responsible for encoding tropomyosin receptor kinase (TRK) fusion proteins [30,36,37]. *NTRK* fusion determines carcinogenic effect in numerous tumors in both mature individuals and juveniles. Patients with DTC and *NTRK* gene fusion have a higher chance of distant metastasis as well as RAI resistance than those with DTC and *BRAF* or *RAS* pathogenic variants. Sequencing of tumor DNA and RNA, and profiling of plasma cell-free DNA are used to detect these fusions [30,36,37].

#### 3.1.3. TERT Promoter Mutation

*TERT*, a ribonucleoprotein polymerase, is capable of lengthening telomeres upon activation. TERT reactivation that is present in many cancers is caused by the alteration of the TERT promoter (*TERTp*). *TERTp* is linked to RAI resistance. Several publications have shown that patients with simultaneously associated *TERT* and *BRAF^V600E^* mutations do not respond to RAI therapy in contrast to patients with only *BRAF^V600E^* pathogenic variant [30,38,39,40,41].

#### 3.1.4. RAS Mutation

MAPK and PI3K cascades are activated by *RAS*. Proto-oncogenes are represented by *NRAS*, *HRAS,* and *KRAS*. Among them, the most common *RAS* genetic alteration remains NRAS codon 61 genetic alteration, proceeding with HRAS codon 61, KRAS codon 12/13, and KRAS codon 61. The association between *RAS* genetic alteration and *BRAF* mutation or *RET/PTC* rearrangement provides a negative prognosis [30,42,43].

#### 3.1.5. ALK Gene Mutation and Fusion

*ALK* gene is known to be a partner in a genetic fusion of t (2;5) chromosome translocation in anaplastic large cell lymphoma. The components of the ALK membrane-binding receptor are represented by extracellular receptor-binding domain, a transmembrane region and an intracellular kinase domain. The mutation or *ALK* gene fusion causes the spontaneous activation of *ALK* leading to the stimulation of MAPK, PI3K-AKT, CRK-like proto-oncogene, CRKL-C3G, MEKK2/3-MEK5-ERK5, and JAK-STAT cascades [30,44,45].

Fusion of *ALK*, which is rare in PTC, was found in thyroid carcinoma through RNA sequencing analysis. Furthermore, a correlation was observed between *ALK* fusion and aggressive thyroid carcinoma. The most common fusion is represented by *ALK* and *STRN* gene. It was found that *STRN-ALK* dimerization leads to *ALK* kinase activation. Thus, targeted therapies on *ALK* fusion are being tried. A novel *ALK* gene fusion, *CCD149-ALK*, was reported using NGS in a woman with RAIR-DTC with disseminated metastasis [30,44,45].

#### 3.1.6. RET Rearrangement

*RET* rearrangement is situated on the long arm of chromosome 10 (10q11.2) and is found in 20% of PTC. It is responsible for encoding TKR of GFL. While *RET* normally contributes to the formation of the kidney and enteric nervous systems during embryogenesis, various factors including ionizing radiation or replication-related stress in DNA fragile sites can lead to DSBs. These breaks can cause *RET* gene fusion, maintaining the kinase domain, which then activates *RET* protein aberrantly. This activation promotes cell proliferation, differentiation, and development through downstream signaling pathways. Importantly, *RET* fusion also affects the production of thyroid cell-specific genes. Consequently, *RET* fusion serves as a carcinogen in PTC, non-small cell lung cancer, and various other malignancies. *RET/PTC1* and *RET/PTC3* rearrangements are the most frequent *RET/PTC* rearrangements [30,46,47,48,49,50,51,52].

#### 3.1.7. PAX8/PPARγ

*PAX8*, which is a component of the transcription factors family, plays a role in promoting the activation of numerous thyroid-specific genes within mature thyroid cells through binding to their promoters. These genes include those that code for thyroglobulin, thyroid peroxidase, and NIS. On the other hand, Peroxisome Proliferator-Activated Receptor Gamma (*PPARγ*), part of the nuclear receptor group of transcription factors, governs systemic fat metabolism and insulin responsiveness [30,53].

The combination of *PAX8* and *PPARγ*, known as *PAX8/PPARγ* rearrangement, arises from a relocation between chromosome regions 2q13 and 3p25. The combination results in the creation of a fusion transcript that codes for *PPFP*. In addition, *PPFP* is found in around 30–35% of FTCs and PTCs [30,53].

Functioning as a cancer-associated protein, *PPFP* can promote cell proliferation, inhibit cell death, as well as enhance DNA replication in the G0/G1 quiescent phase. Notably, the expression of *PPFP* in human thyroid cancer cell cultures modulates the regulation of thyroid-specific genes, including SLC5A5, TPO, TG, and TSHR, that are regulated through PAX8 to different levels. Dysregulation of these PAX8 target sequences as well as their associated pathways is believed to underlie the carcinogenic effects of *PPFP* [30,53].

#### 3.1.8. SWI/SNF Complex Alteration

*SWI/SNF* chromatin remodeling complex alteration is a highly conserved molecular complex comprising 10–15 subunits. It associates with histones and transcription regulators and is categorized into BAF, PBAF, and ncBAF complexes. While these complexes contain shared subunits like SMARCC1/2 and SMARCD1/2/3, they additionally possess specific subunits like ARID1A or ARID1B [30,54,55].

Gene mutations leading to *SWI/SNF* complex deletion result in decreased chromatin accessibility, thereby weakening the regulation of thyroid-specific transcription factors (TF) such as *Foxe1*, *Nkx2–1*, and *PAX8*, crucial for iodization. Deletion of specific subunits like ARID1A, ARID2, or SMARCB1 has been linked to the progression of *BRAF^V600E^*-driven mouse TC. Furthermore, absence of the *SWI/SNF* complex can counteract the treatment efficacy of MAPK blockers and re-differentiation treatments [30,54,55].

### 3.2. Regulation of Signaling Pathways

#### 3.2.1. TSHR Pathway Activation

TSH regulates the regulation of NIS within thyroid follicular cells. TSH links to its receptor (TSHR), a glycoprotein receptor belonging to the G protein-coupled receptor (GPCR) class, located on the cell surface. Stimulation of TSHR through TSH or other signaling factors triggers various G proteins and subsequent pathways, influencing thyroid cell proliferation and the synthesis and hormonal secretion from the thyroid [30,56,57,58,59].

The relationship between TSH and TC cells operates on two levels. From one perspective, it aids therapy by activating pathways like cAMP, promoting activation of thyroid-specific genes like NIS. On the other hand, TSH can stimulate cancer cell growth through pathways like PI3K and MAPK. Moreover, the TSH-TSHR signaling pathway can facilitate immune evasion by tumor cells by inducing expression of tumor PD-L1, suppressing T cell killing effects [30,56,57,58,59].

#### 3.2.2. MAPK Pathway

The MAPK pathway, crucial for regulating thyroid-specific gene expression, is frequently implicated in TC development. The MAPK family comprises ERK, JNK/SAPK, and p38 MAPK, facilitating signal transmission from the extracellular environment to intracellular targets [30,59,60,61,62,63]. In TC, aberrant MAPK pathway activation governs cell division, expansion, and viability. Notably, MAPK activation promotes dedifferentiation of DTC, marked by reduced expression of thyroid hormone production genes including NIS, TPO, and TG, often via downregulation of histone acetylation in NIS gene promoters [30,59,60,61,62,63].

**Figure 1:**
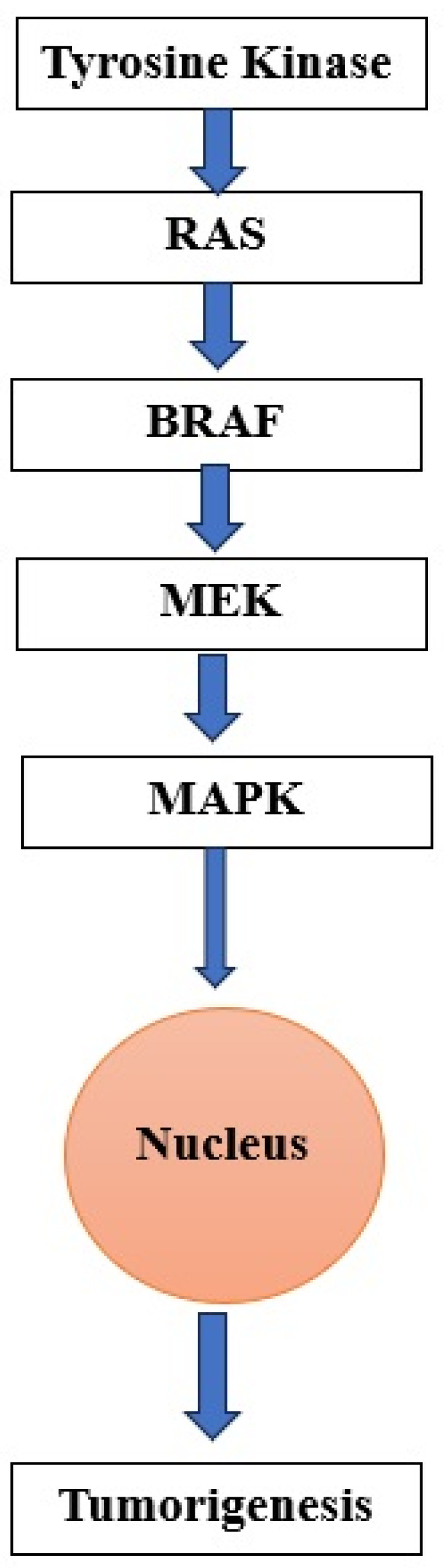
MAPK pathway in PTC [29,30,59,60,61,62,63]. Abbreviations: BRAF—V-Raf mouse sarcoma virus oncogene homologous B1; MEK—mitogen-activated protein kinase/extracellular signal-regulated kinase kinase; MAPK—mitogen-activated protein kinase.

The predominant driver of MAPK pathway perturbation observed in RAIR-DTC is the BRAF^V600E^ pathogenic variant, complemented by a spectrum of BRAF genetic alterations, RAS genetic alterations, as well as mutations in the MEK gene [29,30,59,60,61,62,63] (Figure 1).

#### 3.2.3. PI3K Cascade

The PI3K signaling pathway contributes significantly to the development of RAIR-DTC, governing critical cellular processes including cellular growth, differentiation, and metastasis in TC. Comprising PI3K, AKT, and mTOR, activation of this pathway, along with the cAMP-independent pathway, counteracts the cAMP-dependent pathway’s promotion of thyroid-specific protein expression like NIS [30,64,65].

Besides the RAS genetic alteration and phospholipase C activation, IGF-2 has a major impact on PI3K initiation. RAIR-DTC cells exhibit high expression levels of IGF-2 and its receptor IR-A. While there are no specific inhibitors for IR-A, dynamic interaction between insulin/IGF systems and discoid-domain-containing receptor (DDRs) has been found. Inhibition or decrease in DDR1 expression notably reduces IR-A and IGF-2 production, causing an elevation in thyroid-related gene expression [30,66,67].

AKT activation drives mTOR signaling and simultaneously alters RUNX2 by phosphorylation. RUNX2 modulates various cellular processes including chondrocyte proliferation, maturation, as well as hypertrophy in endochondral ossification. Furthermore, it governs gene activity implicated in TC progression, infiltration, and metastasis. Excessive AKT activity leads to increased expression patterns and transcriptional processes of RUNX2, subsequently amplifying PI3K, AKT, and mTOR expression. This interplay between PI3K/AKT/mTOR cascade and RUNX2 significantly drives cancer growth. MAPK4, a distinct MAPK, can activate AKT through binding to it directly and promoting phosphorylation at threonine 308. Additionally, MAPK4 activates mTORC2, facilitating serine 473 phosphorylation of AKT. Hence, targeting MAPK4 may provide a novel treatment option for RAIR-DTC [30,68,69,70,71].

The mTOR protein, situated under the influence of the PI3K/AKT signaling cascade, works as a serine-threonine protein kinase crucial for regulating various cellular activities such as metabolism, cell division, and longevity, alongside modulating gene expression of key thyroid factors including NIS, essential for RAI uptake. Research has shown that suppression of mTOR with an mTOR inhibitor enhances iodine absorption in TSH-stimulated PCCL3-derived cells from thyroid. However, the impact of rapamycin on iodine uptake appears to be less pronounced compared to inhibition of PI3K, suggesting that mTOR regulates both cell survival and the iodine absorption capacity of thyroid cells [30,72].

#### 3.2.4. TGF-β Pathway

Aberrant TGF-β signaling is linked to multiple diseases, particularly cancer. In human thyroid malignancies, TGF-β is upregulated and serves as a strong promoter in tumor formation and metastasis. In PTC, the *BRAF^V600E^* alteration stimulates active TGF-β1, initiating TGF-β-induced autocrine loop. This mutation also increases levels of both total and phosphorylated Smad3. Initiation of the TGF-β/Smad signaling pathway enhances NOX4 gene expression, which, in turn, forms a heterodimeric complex with p22phox, a regulatory subunit of NOX. This complex regulates the TGF-β/Smad3 cascade by generating ROS. ROS generated by NOX4 act as second messengers, suppressing the progression of TC, especially the expression of NIS, while promoting their proliferation and metastasis. Thus, *BRAF^V600E^*-induced RAIR-DTC is significantly influenced by the TGF-β/Smad signaling pathway [30,73,74].

#### 3.2.5. Wnt/β-Catenin Pathway

Wnt glycoproteins release the transcription factor β-catenin from a protein complex by interacting with Frizzled and LDL receptor-related proteins. This prevents β-catenin’s phosphorylation and degradation, allowing it to enter the nucleus and regulate gene expression by binding to T cell factor (TCF) [30,75,76]. The Wnt/β-catenin pathway significantly influences TC growth and differentiation. In cancer stem cells (CSCs), β-catenin is upregulated, enhancing CSC self-renewal and proliferation, which drives TC progression. Increased lysine-specific histone demethylase 1A (LSD1) in CSCs upregulates β-catenin by downregulating adenomatous polyposis coli 2 (APC2) and Dickkopf-related protein 1 (DKK1), both of which normally promote β-catenin degradation. This pathway activation increases CSCs and contributes to TC’s chemotherapy resistance [30,77,78].

The Wnt/β-catenin signaling supports TC cell proliferation with *BRAF^V600E^* mutations. Knocking out β-catenin slows tumor growth and reduces papillary structures. Additionally, treatment with PKF118-310, a β-catenin-specific inhibitor, enhances the responsiveness of these cancer cells to the BRAFV600E inhibitor PLX4720, leading to substantial growth arrest, cell apoptosis in vitro, and tumor regression and differentiation in vivo [30,79,80].

The β-catenin pathway’s activation can cause disrupted membrane targeting of NIS, contributing significantly to 131I resistance in thyroid cancer cells [30,81].

#### 3.2.6. Notch-Related Pathway

The Notch receptor functions as a multifunctional transmembrane protein and is involved in regulating cell maturation, development, replication, and survival. Humans possess four Notch receptors (Notch1-4) and five ligands (δ-like 1, 3, 4, and Jagged-1, -2). When a Notch receptor interacts with its ligand, it undergoes cleavage by the γ-secretase protease complex, which releases a cytoplasmic segment that moves into the nucleus to modulate gene transcription [30,82,83,84].

In DTC, the levels of Notch receptors and other components of the Notch signaling pathway are markedly reduced in comparison to normal thyroid tissue. Increased expression of Notch receptors in DTC can induce them to regain differentiation by enhancing thyroid-specific genes such as NIS and TPO. Additionally, Notch can reduce cancer cell growth and proliferation rates. Therefore, Notch acts as a crucial controller of thyroid-specific genes and a tumor suppressor in DTC cells [30,82,85,86,87].

### 3.3. Modulation of microRNAs

MicroRNAs (*miRNAs*) are small, unpaired noncoding RNAs that influence gene expression by attaching to the 3′-untranslated region (3′-UTR) of target mRNAs, disrupting their integrity and inhibiting molecular translation [30,88].

Several *miRNAs*, including *miRNA-146b-3p* as well as *miRNA-339*, regulate NIS expression in PTCs by binding to *NIS mRNA 3′-UTR*. *MiRNAs* such as *miRNA-339-5p* and *miRNA-195* also impact RAI uptake in PTCs, with *miRNA-339-5p* being moderately increased and *miRNA-195* significantly decreased in these cancers. Additionally, *miRNA-146b-3p* disrupts RAI uptake by binding to *PAX8* and *NIS mRNA*, contributing to cancer cell proliferation and migration while inhibiting apoptosis. Further *miRNAs*, such as *miRNA-106a*, *miRNA-let-7*, and *miRNA-875*, reduce NIS expression or affect its membrane localization, promoting dedifferentiation in TC. Targeting these *miRNAs* to improve RAI uptake and NIS expression offers a potential therapeutic strategy for TC [30,88,89,90,91,92,93,94,95,96].

## 4. Management of RAIR-DTC

### 4.1. Monitoring

RAIR-DTC is asymptomatic for years. Thus, a careful clinical and laboratory assessment should be performed. Every patient with metastatic TC depends on thyroid function regulation to keep the TSH value suppressed. Therefore, laboratory evaluations ought to involve TSH, fT4, and calcium level post-surgery hypoparathyroidism every 6–12 months. Tumor burden can be evaluated using Tg levels, knowing that Tg doubling time under one year indicates negative predicted outcome and suggests rapid progression of the disease [1,27,97,98,99,100].

Regular imaging every 6 to 12 months using CT scanning and implementing RECIST criteria helps to evaluate the growth of neoplastic mass. Additionally, 18-FDG-PET/CT scanning may provide prognostic indicators in advanced TC. According to studies, patients with lesions with increased glucose uptake have negative prognoses and shorter survival than patients with FDG-PET-negative tumor lesions. The extension of local tumor as well as complications can also be appreciated by other imaging techniques such as bronchoscopy or esophagoduodenoscopy [1,27,97,98,99,100].

### 4.2. Local Treatments

In order to sustain the patients’ standard of living, before starting targeted therapies with tyrosine kinase inhibitors (TKIs), a complete anamnesis regarding age, medical history, size, position, and rate of lesion progression should be completed. Surgery, including the dissection of the central and lateral regions, remains the standard in therapeutic management of locoregional relapse every time the surgical procedure can be safely performed amid re-intervention or distant spreading of the originating malignancy [101,102,103]. Studies have demonstrated that surgery and external-beam radiation treatment (EBRT) in doses of 40–50 Gy for patients older than 45 years, offers a locoregional control and an overall good prognosis in most cases. Local therapies are recommended before targeted therapies for patients with lung nodules or bone metastasis [1,27,97,98,99,100].

In case of infiltration of the trachea, ablative laser therapy should be performed in order to reduce the obstruction. This treatment can be repeated every 6 months. In case of a compression of the trachea as a result of the local tumor mass, an endotracheal stent should be used. Surgery is essential for the resection of bone and lung metastases [1,27,97,99,101].

Depending on the evolution of the TC and the behavior of metastases, percutaneous interventional techniques may be vascular, ablative, or consolidative treatments. Trans-arterial chemoembolization (TACE) is a vascular technique, and it is part of the category of palliative therapies for both advanced hepatocellular cancers and aggressive TCs. This procedure is used in the case of metastases that do not exceed 3 cm and with liver damage of less than 30% [1,104,105].

Radiofrequency thermoablation uses electromagnetic waves that cause movement and heating of the tumor cells. In other words, the technique is used to reduce the volume of the metastatic lesion in the case of metastases involving the lymph nodes, bones, liver, and lung [1,27,106,107,108].

Lymph nodes with metastases smaller than 1 cm may be monitored periodically every 6 months and if they increase in size, ultrasound-guided percutaneous ethanol ablation can be performed. For bone metastases with osteolytic lesions, combinations of local and palliative treatments such as cementoplasty can enhance the patient’s quality of life by alleviating pain and ensuring bone stability [1,27,109].

### 4.3. TKIs as Targeted Therapies

**Figure 2:**
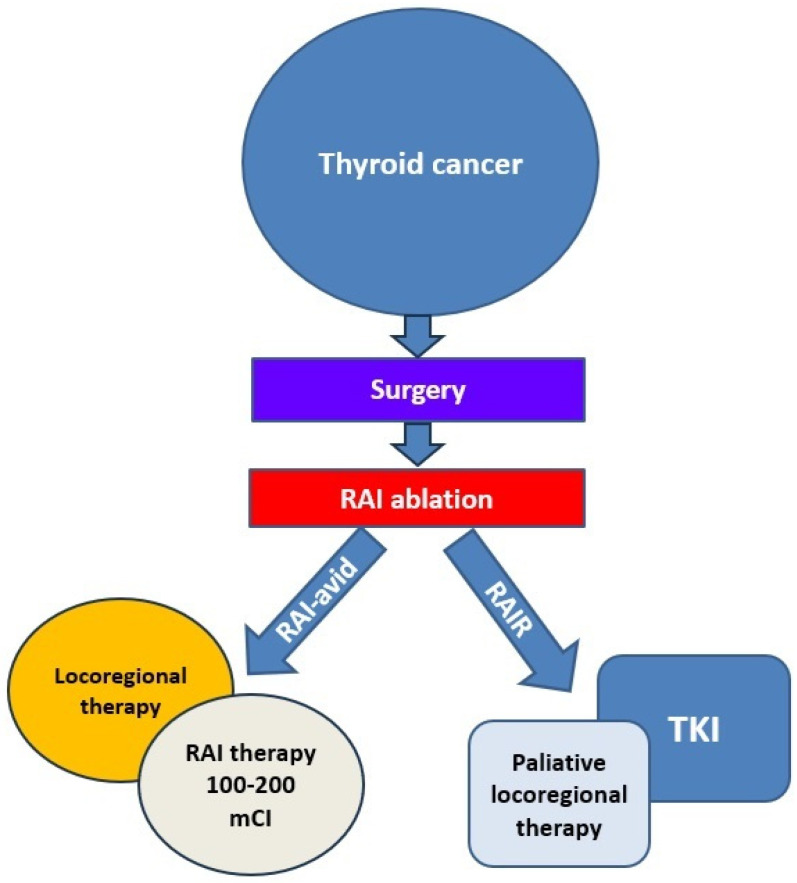
Treatment approach for advanced/metastatic DTC [1,28,30]. Abbreviations: RAI—radioactive iodine; RAI-avid—radioactive iodine-avid; TKI—tyrosine kinase inhibitor.

Since RAIR-DTC does not respond to RAI due to the previously presented mechanisms, clinical trials and preclinical studies are being conducted with new drugs that would be successful in treating these patients. Currently, TKIs are now considered the first therapeutic line to inhibit the expansion and progression of RAIR-DTC [1,28,30] (Figure 2).

**Figure 3:**
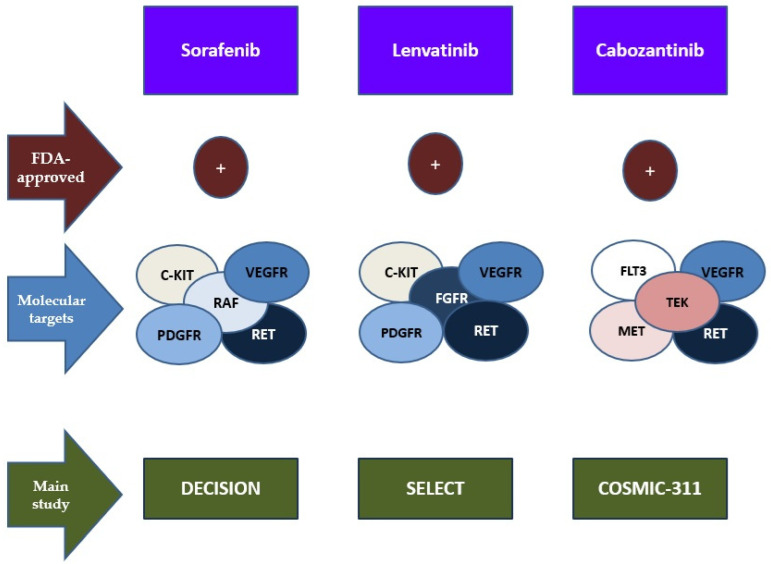
FDA-approved TKIs for RAIR-DTC [112,121,123]. Abbreviations: FDA—Food and Drug Administration; c-MET—hepatocyte growth factor receptor or HGFR; c-KIT—stem cell factor receptor or SCFR; EGFR—epidermal growth factor receptor; FGFR—fibroblast growth factor receptor; FLT3—FMS-like tyrosine kinase 3 (or CD135); PDGFR—platelet-derived growth factor receptor; RET—ret proto-oncogene; RAF—rapidly accelerated fibrosarcoma; VEGFR—vascular endothelial growth factor receptor; DECISION ClinicalTrials.gov number, NCT00984282; SELECT ClinicalTrials.gov number, NCT01321554; COSMIC-311 ClinicalTrials.gov number NCT03690388.

**Figure 4:**
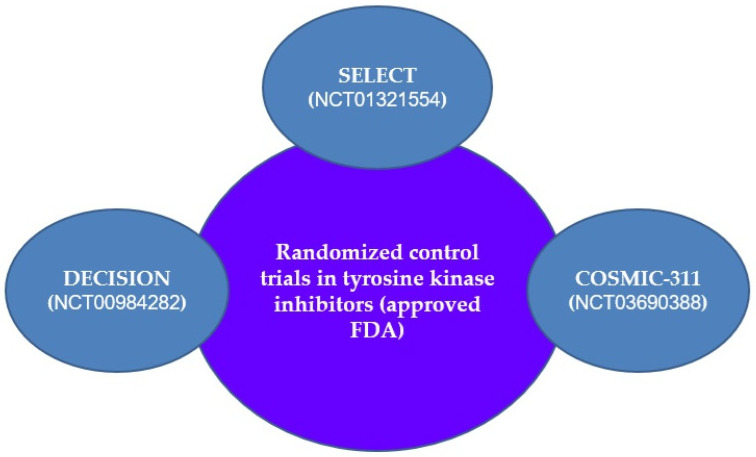
Randomized control trial of FDA-approved TKIs for RAIR-DTC [112,121,123]. Abbreviations: FDA—Food and Drug Administration; DECISION ClinicalTrials.gov number, NCT00984282; SELECT ClinicalTrials.gov number, NCT01321554; COSMIC-311 ClinicalTrials.gov number NCT03690388.

Sorafenib, lenvatinib, and cabozantinib have been approved by the US Food and Drug Administration (FDA) for treating RAIR-DTC [1,30,100] (Figure 3 and Figure 4).

Sorafenib targets RAF and blocks VEGFR1/2/3, c-KIT, RET, PDGFR, and FLT receptors. In the phase 3 DECISION trial, 417 subjects with advanced or metastatic DTC who had progressive RAIR disease were administered 400 mg of sorafenib, taken twice a day. The trial showed that 12.2% of patients receiving sorafenib achieved a partial response (PR), compared to just 0.5% in the placebo group. Progression-free survival (PFS) improved from 5.8 months to 10.8 months, while overall survival (OS) remained stable. Notably, 78% of subjects needed dose modifications due to side effects [1,4,28,30,110].

Lenvatinib (E7080), a multi-kinase oral inhibitor that targets VEGFR, FGFR, PDGFRα, RET, and KIT, was approved by the FDA in 2015 for treating RAIR-DTC. In the phase 3 SELECT trial, lenvatinib significantly improved PFS and response rates compared to placebo in RAIR-DTC patients. A sub-analysis revealed that while lenvatinib improved PFS in both younger and older patients, older patients experienced more toxicity. Despite allowing crossover after disease progression, an OS benefit was noted in older subjects. However, lenvatinib used alone was found to be less effective for treating ATC, warranting further investigation [1,4,28,30,108,109,110,111,112,113,114].

A phase 3 study (NCT02966093) was performed across 24 sites in China to investigate the safety and efficacy of lenvatinib in treating RAIR-DTC in this population. The results showed that a starting dose of 24 mg/day led to a significant improvement in PFS and objective response rates compared to placebo, with no new or unexpected side effects reported. These findings are consistent with the SELECT trial results [1,4,28,30,111,112,113,114,115,116,117].

Additionally, a correlation was found between lung metastases in RAIR-DTC patients and reduced survival rates. A post hoc analysis by Tahara et al. (2021) of the SELECT data indicated that lenvatinib led to improved OS in patients with lung metastases greater than or equal to 1.0 cm, despite a crossover rate of 89%. Prompt initiation of treatment can enhance outcomes in these patients [1,4,28,30,111,112,113,114,115,116,117].

While lenvatinib’s toxicity is usually manageable through dose adjustments, Tahara et al. (2019) noted that shorter treatment interruptions were associated with better outcomes. This emphasizes the significance of early management of lenvatinib-related side effects to optimize its effectiveness in RAIR-DTC subjects [1,4,28,30,111,112,113,114,115,116,117].

Cabozantinib is an inhibitor targeting c-MET, RET, and VEGFR that has received FDA approval for MTC after the phase 3 trial, demonstrating a 7.2-month rise in median PFS. Initial phase 1 studies revealed a 62% objective response rate in eight subjects with DTC who had undergone prior VEGFR-targeted treatment [1,4,28,30,118,119,120,121,122].

Building on these encouraging findings, a phase 2 study highlighted cabozantinib’s efficacy in subjects with RAIR-DTC that had disease progression after previous treatments. A later phase 3 trial further confirmed that cabozantinib significantly improved PFS among RAIR-DTC subjects that lacked conventional treatment options [1,4,28,30,118,119,120,121,122].

On 17 September 2021, cabozantinib received FDA approval for use in adults and pediatric patients 12 years and older with locally advanced or metastatic DTC who showed progression following previous VEGFR-targeted therapy. This approval marked a significant advancement in treatment options, providing hope for patients facing limited alternatives and demonstrating the viability of cabozantinib in treating RAIR-DTC. Additionally, ongoing research is expected to further elucidate the long-term effects and potential combination therapies involving cabozantinib to enhance outcomes for patients with this challenging disease [1,4,28,30,118,119,120,121,122].

Vandetanib is an inhibitor that targets multiple pathways, including VEGFR2/3, EGFR, c-KIT, and RET. Although its use in treating RAIR-DTC has not yet received approval, an earlier phase 2 randomized trial indicated a favorable response in this subject population, with a median PFS of 11.1 months in the vandetanib group, versus 5.9 months in the placebo group. A phase 3 trial (VERIFY) was completed in 2020 with 119 patients suffering from progressive RAIR-DTCs. The preliminary data indicated no significant difference in PFS between the vandetanib and placebo groups (10 months vs. 5.7 months, *p* = 0.08) (NCT01876784, ClinicalTrials.gov accessed on 20 August 2024) [1,4,28,30,123].

Long-term use of TKIs in clinical practice can lead to moderate to severe adverse effects, particularly in patients aged 65 and older, necessitating careful monitoring for dosage adjustments [113]. Additionally, research showed that RAIR-TC patients often develop TKI resistance, resulting in “tumor escape” due to alterations in alternative signaling pathways, such as HER2/3 hyper-expression. Nonetheless, a retrospective analysis indicates that RAIR-DTC refractory to initial TKI treatment may continue to show effectiveness with salvage therapies, including candetanib, cabozantinib, sunitinib, pazopanib, and vemurafenib [1,4,28,30,124,125].

Table 2 provides an overview of the targeted kinase inhibitors evaluated in randomized-controlled trials for advanced, metastatic RAIR-DTC.

**Table 2:** FDA-approved TKIs for RAIR-DTC (PubMed search based on key terms “RAIR-DTC”, “tyrosine kinase inhibitors”) [112,121,123].

Randomized Control Trial	Drug	Molecular Targets	Phase	Results: PFS	ORR
DECISION [123]	Sorafenib	VEGFR, PDGFR, c-KIT, RET, RAF	III	from 10.8 months to 5.8 months (placebo)	12.2% (vs. 0.5%)
SELECT [112]	Lenvatinib	VEGFR, PDGFR, c-KIT, RET, FGFR	III	18.3 months vs. 3.6 months (placebo)	64.8% (vs. 1.5)
COSMIC-311 [121]	Cabozantinib	VEGFR, RET, c-MET, FLT3, TEK	III	11.0 months vs. 1.9 months placebo	15%

Abbreviations: PFS—progression-free survival; ORR—overall response rate, c-MET—hepatocyte growth factor receptor or HGFR; c-KIT—stem cell factor receptor or SCFR; EGFR—epidermal growth factor receptor; FGFR—fibroblast growth factor receptor; FLT3—FMS-like tyrosine kinase 3 (or CD135); PDGFR—platelet-derived growth factor receptor; RET—ret proto-oncogene; RAF—rapidly accelerated fibrosarcoma; VEGFR—vascular endothelial growth factor receptor.

### 4.4. Redifferentiation Therapy

In light of the pathophysiology associated with RAI refractoriness, research has focused on re-inducing NIS expression to restore RAI avidity. Inhibitors targeting the MAPK pathway have demonstrated potential in facilitating redifferentiation process in RAIR-DTC. For instance, the MEK inhibitor selumetinib increased RAI avidity in 12 out of 20 RAIR-DTC patients, enabling 8 of these subjects to receive RAI treatment, in which 7 exhibited a partial response; however, information on the duration of these responses is not available [4,126,127].

Additionally, the application of *BRAF* inhibitors, including vemurafenib and dabrafenib, has been investigated in patients with *BRAF*-mutated RAI-R TC, producing similar outcomes. Nevertheless, it has been noted that thyroid cancers with *BRAF* pathogenic variants tend to respond less favorably to redifferentiation therapies, suggesting that a stronger inhibition of the MAPK pathway may be necessary, potentially through a dual therapy of *BRAF* and MEK inhibitors. A short duration of these treatment regimens (typically lasting 4–8 weeks in most studies) may lead to significantly lower toxicity compared to long-term use of multi-kinase inhibitors (MKIs), thus alleviating some of the economic burdens associated with treatment. While redifferentiation therapies appear promising, the current evidence regarding their clinical efficacy remains preliminary, necessitating larger clinical trials to confirm these results [4,126,127,128,129,130,131,132,133,134].

### 4.5. Immunotherapy

The introduction of checkpoint blockade therapies, such as anti-PD-1, PD-L1, and PD-L1-4, marks a significant advancement in treating various tumors. Current research indicates that PD-L1 could be used as a prognostic biomarker for PTC as well as indicate recurrence in MTC. A retrospective analysis identified high PD-L1 level in ATC, correlating with worse overall life expectancy plus PFS, positioning PD-L1 as a possible predictive marker of ATC outcomes. Furthermore, immunotherapy approaches have been studied in subjects with advanced RAIR-DTC [28,135,136,137].

The non-randomized phase Ib KEYNOTE-028 trial (NCT02054806) investigated the effectiveness of pembrolizumab in 22 patients with advanced RAIR-DTC expressing PD-L1. Pembrolizumab, a PD-1 antibody, was administered biweekly at a dose of 10 mg/kg for a maximum of 24 months. Among the participants, two patients (9%) showed a partial response, with durations of response ranging from 8 to 20 months. Median PFS was 7 months, with the median OS yet to be reached. Adverse events occurred in 18 subjects (82%), with gastrointestinal distress and tiredness being the most common [28,138].

A phase I/II trial (NCT02404441) involving 30 ATC patients treated with spartalizumab (400 mg every 4 weeks) demonstrated an overall response rate (ORR) of 17% and disease control in 27%. Common adverse events included gastrointestinal discomfort, such as diarrhea, pruritus, fatigue, and hematologic and oncologic complications. In another phase 2 trial led by Capdevila, 42 patients received spartalizumab (400 mg/month), with an ORR of 19%. PD-L1-positive patients responded better (29% vs. 0%) and those with the *BRAF* pathogenic variant had long-lasting responses, with a 1-year survival rate of 52.1% [28,139,140].

A phase 2 trial assessing pembrolizumab in combination with chemoradiotherapy in three subjects with ATC initially showed favorable tumor reactions, but every patient died within 6 months from metastases or pulmonary disorders, highlighting concerns over the high toxicity of chemoradiotherapy in ATC [28,141]. According to very recent data, PD-L1 expression is not correlated with the response to combined treatment [142,143].

The novelty of the topic is as follows: immunotherapy in radioiodine-refractory thyroid cancer is an emerging and innovative area of research, offering new potential for patient recruitment. Despite its limited accessibility, it provides an additional prognostic approach and underscores the importance of a multidisciplinary team in optimizing patient outcomes. [27,30,144,145].

## 5. Conclusions and Future Perspectives

Despite the generally good prognosis of thyroid tumors, a small portion of subjects with advanced or progressive TC will not respond to radioiodine treatment, which is responsible for the majority of TC-related deaths. Significant efforts have been devoted to understanding the molecular mechanisms behind this, leading to notable advancements in identifying the genetic and epigenetic changes associated with iodine resistance. This progress has facilitated the development of several possible treatments for RAIR-DTC.

Three TKIs are approved for RAIR-DTC treatment, and several more are in clinical trials. However, the considerable toxicity related to these drugs presents serious concerns. Given this risk, the use of TKIs should be restricted to carefully selected patient populations, with thorough evaluations and interdisciplinary input from experienced clinicians required before personalizing treatment or considering clinical trial enrollment.

Redifferentiation therapies, particularly those involving *BRAF* and *MEK* antagonists, have proven notable progress in enhancing responsiveness in RAIR-DTC patients, offering comparable response to TKIs with reduced adverse effects. PD-1/PD-L1 blockade, a key immunotherapeutic approach in oncology, shows promise, but its application in RAIR-DTC is still not well established, requiring larger studies to evaluate its potential.

Looking ahead, the advancement of targeted therapies, such as TKIs, MAPK inhibitors, and checkpoint inhibitors, holds significant promise for RAIR-DTC. Combination therapies targeting different pathways may offer new treatment options, with dual targeting of key molecules like *BRAF* and *MEK* potentially overcoming compensatory mechanisms that lead to drug resistance.

## Figures and Tables

**Table 1:** Inclusion and exclusion criteria.

Inclusion Criteria
Original studies
Topic: gene data, radiodiodine-refractory
Published in PubMed
Timeframe of search: 1998–2024
**Exclusion criteria**
Non-human data
Case report, case series
Editorial
Non-English paper
Pediatric data
Selective inhibitor of RET
Selective inhibitor of NTRK
RAI-avid
MTC

Abbreviations: RAI-avid—radioactive iodine-avid; MTC—medullary thyroid carcinoma.

## Abbreviations

TC	Thyroid cancer
DTC	Differentiated thyroid cancer
RAI	Radioactive iodine
TSH	Thyroid-stimulating hormone
MAPK	Mitogen-activated protein kinase
PI3K/mTOR/Akt	Photoshatidyl-inositol-3-kinase/mammalian target of rapamycin/protein kinase B
BRAF	V-Raf mouse sarcoma virus oncogene homologous B1
TGF-β	Transforming growth factor-β
NIS	Sodium iodine symporter
PTEN	Phosphatase and tensin homolog
ATC	Anaplastic thyroid cancer
TERT	Telomerase reverse transcriptase
RAIR-DTC	Radioiodine-refractory differentiated thyroid cancer
SLC5A5	Solute carrier family 5A
cAMP	Cyclic adenosine monophosphate
NUE	NIS upstream enhances
PKA	Protein kinase A
PAX8	Paired box gene-8
Ref-1	Redox effector factor-1
CREM	aAMP-response element modulator
MAPKKK	Mitogen activated protein kinase kinase kinase
V600E	B-raf protein residue 600 from glutamic acid to valine
AHR	Aromatic hydrocarbon receptor
WT1	Wilm tumor gene 1
NTRK	Neurotrophic receptor tyrosine kinase
TRK	Tropomyosin receptor kinase
TERTp	TERT promoter
ALK	Anaplastic lymphoma kinase
CRKL-C3G	Adaptor protein-Rap guanine nucleotide exchange factor 1
MEKK2/3-MEK5-ERK5	Mitogen-activated protein kinase kinase kinase 2/3-Mitogen-activated protein kinase kinase 5-extracellular signal regulated kinase 5
JAK-STAT	Janus linase-signal transducer and activator of transcription
STRN	Recurrent striatal protein
NGS	Next-generation sequencing
TKR	Tyrosine kinase membrane receptor
GDNK	Glial cell line-derived neurotrophic factor
GFL	GDNF family ligand
DSBs	DNA double-strand breaks
PPAR-γ	Peroxisome proliferator-activated receptor gamma
PPFP	PAX8-PPAR-γ fusion protein
TPO	Thyroid peroxidase
TG	Thyroglobulin
TSHR	Thyroid-stimulating hormone receptor
SWI/SNF	SWItch/sucrose nonfermentable
BAF	BRG1/BRM related factor
PBAF	Polybromine-related factor
ncBAf	Atypical BAF
TF	Transcription factors
GPCR	G protein-coupled receptor
PD-L1	Tumor programmed death-ligand 1
ERK	Extracellular-signal-regulated kinase
JNK/SAPK	Jun kinase
MEK	Mitogen-activated protein kinase/extracellular signal-regulated kinase kinase
IGF-2	Insulin-like growth factor 2
IR-A	Insulin receptor subtype A
IIGFs	Insulin/insulin-like growth factor systems
DDRs	Discoid domain receptors
RUNX2	Runt-related transcription factor 2
mTORC2	mTOR complex 2
NADPH	Nicotinamide adenine dinucleotide phosphate
NOX4	NADPH oxidase 4
ROS	Reactive oxygen species
TCF	T cell factor
CSCs	Cancer stem cells
LSD1	Lysine-specific histone demethylase 1 A
APC2	Adenomatous polyposis coli 2
DKK1	Dickkopf-related protein 1
Notch1-4	Notch receptors
miRNAs	MicroRNAs
3′-UTR	3′-untranslated region
fT4	Free thyroxine
TKIs	Tyrosine kinase inhibitors
EBRT	External-beam radiation therapy
TACE	Trans-arterial chemoembolization
VEGFR1/2/3	Vascular endothelial growth factor receptor 1, 2, 3
c-KIT	Cellular kit
PDGFR	Platelet-derived growth factor receptor
FLT	Fms-like tyrosine kinase
FDA	Food and Drug Administration
PR	Partial response
PFS	Progression-free survival
OS	Overall survival
FGFR	Fibroblast growth factor receptor
c-MET	Mesenchymal–epithelial transition factor
MTC	Medullary thyroid carcinoma
EGFR	Epidermal growth factor receptor
ORR	Overall response rate
RAF	Rapidly accelerated fibrosarcoma
MKIs	Multi-kinase inhibitors
CTLA-4	Cytotoxic T-lymphocyte antigen 4
